# Alterations in Maternal–Fetal Heart Rate Coupling Strength and Directions in Abnormal Fetuses

**DOI:** 10.3389/fphys.2019.00482

**Published:** 2019-04-26

**Authors:** Ahsan H. Khandoker, Steffen Schulz, Haitham M. Al-Angari, Andreas Voss, Yoshitaka Kimura

**Affiliations:** ^1^Department of Biomedical Engineering, Healthcare Engineering Innovation Center, Khalifa University of Science and Technology, Abu Dhabi, United Arab Emirates; ^2^Institute of Innovative Health Technologies IGHT, Ernst-Abbe-Hochschule, Jena, Germany; ^3^Institute of International Advanced Interdisciplinary Research, Tohoku University School of Medicine, Sendai, Japan; ^4^Department of Gynecology and Obstetrics, Tohoku University Hospital, Sendai, Japan

**Keywords:** fetal heart rate, maternal heart rate, coupling, partial directed coherence, Granger causality

## Abstract

Because fetal gas exchange takes place via the maternal placenta, there has been growing interests in investigating the patterns and directions of maternal-fetal cardiac coupling to better understand the mechanisms of placental gas transfer. We recently reported the evidence of short-term maternal–fetal cardiac couplings in normal fetuses by using Normalized Short Time Partial Directed Coherence (NSTPDC) technique. Our results have shown weakening of coupling from fetal heart rate to maternal heart rate as the fetal development progresses while the influence from maternal to fetal heart rate coupling behaves oppositely as it shows increasing coupling strength that reaches its maximum at mid gestation. The aim of this study is to test if maternal-fetal coupling patterns change in various types of abnormal cases of pregnancies. We applied NSTPDC on simultaneously recorded fetal and maternal beat-by-beat heart rates collected from fetal and maternal ECG signals of 66 normal and 19 abnormal pregnancies. NSTPDC fetal-to-maternal coupling analyses revealed significant differences between the normal and abnormal cases (normal: normalized factor (NF) = −0.21 ± 0.85, fetus-to-mother coupling area (A_fBBI→ mBBI) = 0.44 ± 0.13, mother-to-fetus coupling area (A_mBBI→ fBBI) = 0.46 ± 0.12; abnormal: NF = −1.66 ± 0.77, A_fBBI→ mBBI = 0.08 ± 0.12, A_mBBI→ fBBI = 0.66 ± 0.24; *p* < 0.01). In conclusion, maternal-fetal cardiac coupling strength and direction and their associations with regulatory mechanisms (patterns) of developing autonomic nervous system function could be novel clinical markers of healthy prenatal development and its deviation. However, further research is required on larger samples of abnormal cases.

## Introduction

Fetal development *in utero* is crucially linked with adequate placental blood circulation because oxygenated blood is transferred across placenta to fetal circulation through umbilical cord from maternal cardiovascular system (Wang, 2010). During each of fetal cardiac cycle, oxygenated blood to fetal heart enters through inferior vena cava instead of pulmonary veins. Therefore, influences of maternal psychological and physiological conditions were investigated by various studies (Bekedam et al., 1991; Jensen, 1996; Welberg and Seckl, 2001). For example, reduced maternal blood oxygen level was reported to have caused hypoxia in fetus (Jensen, 1996). Maternal exercise with a moderate increase in maternal heart rate caused elevation in fetal heart rate (Webb et al., 1994). Increased maternal stress and anxiety levels were also reported to be associated with increased fetal heart rate (Monk et al., 2000). Fetal–maternal heart rate phase synchronization was also investigated in different settings, including controlled maternal respiration and maternal aerobic exercise (Van Leeuwen et al., 2009, 2014). Results of those studies suggested that high maternal breathing rate might induce the synchronization as it occurred significantly more often at fast maternal breathing and less at slow respiratory rates (Van Leeuwen et al., 2009). Synchronization was found less often where mothers had exercised regularly, possibly due to an increased beat-to-beat differences, higher vagal tone and slower breathing rates (Van Leeuwen et al., 2014). It was also suggested that the short time fetal–maternal heart rate coupling might occur through auditory stimulation associated with the maternal heart rhythms, perceived by the fetal auditory pathways (Riedl et al., 2009; Van Leeuwen et al., 2014). However, determination of the underlying mechanisms and patterns required further investigation of the coupling and its directionality (fetal to maternal and vice versa).

Given those evidences, mathematical methods to investigate and quantify the interactions between maternal and fetal cardiac systems were proposed in several research papers (Riedl et al., 2009; Van Leeuwen et al., 2009, 2014). As such, the analyses of causal and non-causal relationships within and between dynamic systems have become increasingly popular especially in physiology when the understanding of cause-response relationships and their directionalities between physiological regulatory system and its subsystems is important (Porta and Faes, 2013; Schulz et al., 2013; Schulz and Voss, 2014). For examples, the characterization of linear and non-linear couplings of the cardiovascular-, cardiorespiratory-, and central regulatory networks were reported by several studies (Schulz et al., 2013; Bartsch et al., 2015; Faes et al., 2015; Ivanov et al., 2016) based on Granger causality; non-linear prediction; entropies; symbolic dynamics and phase synchronization. Furthermore, more new concepts such as time delay stability (TDS) approach to study different physiological interactions among systems with different dynamics (Liu et al., 2015), TDS approach (Liu et al., 2015), coupling functions (Stankovski, 2017), dynamical causal modeling (Friston et al., 2003), and bispectrum (Siu et al., 2008; Schulz et al., 2018); were also reported. Among the Granger causality based approaches in frequency domain, Partial Directed Coherence (PDC) and the directed transfer function (DTF), and their enhanced versions (e.g., the normalized short time partial directed coherence (NSTPDC) are the most recent techniques applied to the oscillatory nature of physiological variables such as beat to beat heart rate (Porta and Faes, 2013). The major limitation of PDC is that it cannot be directly used for non-stationary signals (Baccalá and Sameshima, 2001). Therefore, a time-variant version of PDC (tvPDC) was proposed to provide information about the partial correlative interaction properties which made it applicable for modeling short-time interactions in cardiovascular systems (Milde et al., 2011). NSTPDC which is one of the tvPDC approaches was developed to evaluate dynamical changes of couplings and applied for detecting the level and direction of couplings in multivariate and complex dynamic systems (Adochiei et al., 2013). In our previous study (Khandoker et al., 2016), NSTPDC was applied to investigate the strength of the directional relationship between fetal–maternal cardiac systems in normal fetuses. In that study we reported that a causal influence of fetal heart rate on maternal heart rate significantly decreased from early to mid-gestation age along with a significant increase of maternal to fetal heart rate. The causal influence of maternal on fetal heart rate was the strongest in the mid gestation and remained dominant in the late gestation of developing autonomic nervous system function (Khandoker et al., 2016). However, it still remains to clarify the nature and directionality of fetal maternal cardiac interaction in sick fetuses. We hypothesize that the strength and directionality of fetal–maternal heart rate couplings in sick fetuses are altered depending on the types of abnormalities and thus could become useful markers in screening abnormal fetuses from normal ones. Therefore, the aim of this study was to evaluate fetal maternal heart rate coupling strength and directions of a variety of abnormal fetuses by using NSTPDC method and the compare the results with the same from normal cases.

## Materials and Methods

Maternal and abdominal ECG were collected from 85 pregnant women at Tohoku University Hospital. Out of them 66 pregnant women were at the gestational age of 16–39 (28.9 ± 6.3) weeks with normal singleton pregnancy and 19 women had abnormal pregnancy at the gestational age of 19–38 (30.3 ± 5.7) weeks. The normal fetuses were selected as per Intrapartum Fetal Monitoring Guidelines (FIGO) (https://www.figo.org/news/available-view-figo-intrapartum-fetal-monitoring-guidelines-0015088). The abnormal group has a variety of abnormalities such as: fetal bradycardia, fetal tachycardia, premature atrial contraction, and different types of congenital heart defects (CHD) or anomalies (ventriculoseptal defect (VSD), atrial septal defect (ASD), pulmonary atresia (PA), tetralogy of Fallot (TOF), and Ebstain anomaly). Demographics of normal and abnormal cases are summarized in Tables 1, 2. The abdominal ECG signals were recorded by using 12 electrodes: 10 on the mother's abdomen, one reference electrode on the back and one electrode at the right thoracic position. Simultaneously the Doppler ultrasound signal from 1.5 MHz Ultrasonic Transducer 5,700 placed on the lower abdomen, was collected as a reference together with the abdominal ECG by a multichannel data acquisition system (fetal monitor 116, Corometrics Medical Systems Inc.). All recordings (each of 1 min length) were collected during daytime (between 9 am and 2 pm) and sampled at 1,000 Hz with 16-bit resolution. The study protocol was approved by Tohoku University Institutional Review Board and written informed consent was obtained from all subjects. Fetal ECG traces were extracted from abdominal ECG signals by using a method that combines cancellation of the mother's ECG signal and the blind source separation with reference (BSSR) as described in our earlier study (Sato et al., 2007). RR intervals (RRI) were determined using the algorithm developed by Pan and Tompkins (Pan and Tompkins, 1985). Two RRI time series namely fetal heart rates (fBBI) and maternal heart rates (mBBI) were extracted from maternal and fetal ECG signals. Both time series were visually inspected and if appropriate reedited. These time series (fBBI, mBBI) were subsequently filtered by an adaptive filter algorithm to remove and interpolate ventricular premature beats and artifacts to obtain normal-to-normal (NN) beat time series. The relative number of excluded beats (RR-intervals) was lower than 5% in relation to the relative duration of all RR intervals in the time series. For the maternal–fetal coupling analyses the filtered fBBI and mBBI time series were resampled (spline interpolation) using synchronization frequency *fs* = 5 Hz, to obtain synchronized time series (300 samples).

**Table 1:** Demographics data for the normal cases.

**Group**	**Gestational weeks**	**Maternal age (years)**	**Maternal BMI**	**EFBW**	**Para**	**Gravida**
GA1(*n =* 22)	21.8 ± 2.4	31.1 ± 3.4	22.6 ± 2.6	654.4 ± 248.9	1.6 ± 1.0	0.7 ± 1.0
GA2(*n =* 22)	29.0 ± 1.3	30.6 ± 3.1	23.0 ± 3.1	1285 ± 291.7	1.2 ± 0.8	0.4 ± 0.5
GA3(*n =* 22)	36.2 ± 1.8	29.9 ± 6.2	22.6 ± 6.1	2571 ± 308.8	0.7 ± 0.8	0.1 ± 0.3

*EFBW, Estimated Fetal Birth Weight; Gravida, number of pregnancy times; Para, number of pregnancies reach viable gestational age; GA1, early gestational age (≤25 weeks); GA2, middle gestational age (26–30 weeks); GA3, late gestational age (≥32 weeks)*.

**Table 2:** Demographics data for the abnormal cases.

**ID**	**Gestational weeks**	**Maternal age (years)**	**Maternal BMI**	**EFBW**	**Para**	**Gravida**	**Delivery mode**	**Abnormality**
1	36	24	23.1	2,373	1	1		WPW
2	25	34	36.2	449	0	0	C–section	Placental dysfunction
3	19	–	–	–	–	–	–	TTTS Donner
4	29	30	22.8	–	–	–	–	Hydrops fetalis
5	31	30	21.8	–	–	3	–	Medical history of intrauterine fetal death
6	36	34	21.1	2,448	1	1	Natural	Fetal Tachycardia
7	38	44	23.4	–	–	–	–	SSS
8	27	–	–	–	–	–	–	AV Block, CHD, SA, CAV
9	34	23	22.1	2,600	0	0	Emergency C–section	Cardiac dilatation, CHD
10	38	26	23.6	–	0	0	C–section	PAC, IUGR
11	37	–	–	–	–	–	–	CHD
12	28	41	22.9	859	1	0	–	TOF, VSD, PA, MS, PAC
13	33	28	25.5	–	–	–	–	Fetal Tachycardia
14	23	36	21.5	–	–	3	–	CHD
15	35	32		2,100	1	1	–	CHD
16	31	37	21.5	1,157	–	–	–	Heart Failure
17	25	41	36.2	767	1	1	–	PA, CAVC, SA, AV Block, Polysplenia syndrome
18	23	25	22.1	–	–	–	–	VSD, ASD, CDH, Chromosomal aberration
19	27	25	22.9	1,332	0	0	stillbirth	Ebstein syndrome

*EFBW, Estimated Fetal Birth Weight; Gravida, number of pregnancy times; Para, number of pregnancies reach viable gestational age. Abnormality abbreviations: WPW, Wolff–Parkinson–White syndrome; TTTS, twin-twin transfusion syndrome; SSS, sick sinus syndrome; CHD, congenital heart defect; SA, single atrium; CAV, cardiac allograft vasculopathy; PAC, premature atrial contraction; IUGR, intrauterine growth restriction; TOF, tetralogy of Fallot; VSD, ventriculoseptal defect; PA, pulmonary atresia; MS, mitral stenosis; CAVC, common atrioventricular canal; ASD, atrial septal defect; CDH, congenital diaphragmatic hernia*.

### Heart Rate Variability Analysis

For the quantification of heart rate variability (HRV) we calculated some of the most commonly used indices in cardiovascular variability analysis in the time domain (TD), the frequency domain (FD) and from non-linear dynamics (NLD) (Voss et al., 2009) from the fBBI time series. These features were:

#### Linear Measures

(1) **meanHR:** mean value of normal fetal heart beats in bpm.
(2) **sdNN:** standard deviation of the NN-intervals of fBBI in ms.
(3) **RMSSD:** root mean square of successive differences of fBBI NN-intervals (ms).
(4) **pNN50:** percentage derived by dividing the number of interval differences of successive NN intervals > 50 ms by the total number of NN intervals.
(5) **pNNI30:** percentage of NN intervals differences < 30 ms.
(6) **Shannon:** Shannon entropy of the fBBI histogram which was defined on the probability distribution of histogram bins. For our analysis, the total number of bins was chosen to be 225 with a width of 8 ms for the range of 200–2,000 ms.
(7) **Renyi025:** Renyi entropy which is a generalization of the Shannon entropy, was computed on the probability distribution similar to Shannon entropy using the following equation:
  (1) $$H_{renyi}\left(a\right)=\frac{1}{1-a}log_{\;2}\sum_{i=1}^{k}p_{i}^{\alpha },\;\alpha >0,\;\alpha \neq 1$$
  For Renyi25, α = 0.25 where α is a weighting factor, *p*_*i*_ is the probability of the *i*-th bin of the fBBI histogram and *k* is the total number of bins which was set to 225 with a width of 8 ms for the range of 200–2,000 ms.

#### Non-linear Measures

(8) **plvar10:** portion of low-variability patterns within the fBBI NN-intervals <10 ms (Kurths et al., 1995; Voss et al., 1996).
(9) **phvar10:** portion of high-variability patterns within the fBBI NN-intervals >10 ms.
(10) **hLZ77w3b3:** Compression entropy with buffer size *b* = 3 and the window length *w* = 5. Compression entropy quantifies to what extent data can be compressed (Baumert et al., 2004). The lower the compression rate the higher the complexity.

### Normalized Short-Time Partial Directed Coherence

To quantify the central autonomic network related to the causal coupling between the CNS- and ANS time series, the NSTPDC approach was applied (Adochiei et al., 2013; Schulz et al., 2015, 2016). It is based on a multivariate autoregressive (AR) model with order *p* to determine linear Granger causality in the frequency domain. NSTPDC is based on the time-variant partial directed coherence approach (tvPDC, π_*xy*_(*f, n*)) providing information about the partial correlative short-time interaction properties of non-stationary signals, with *f* as the frequency and *n* the number of windows (Milde et al., 2011).

For the selection of the optimal order *p* of the AR(*p*) model the stepwise least squares algorithm and the Schwarz's Bayesian Criterion (SBC) were used. To quantify the coupling direction between two time series, *x* and *y* (mBBI and fBBI: with *x*_mBBI_ and *y*_fBBI_) a coupling factor (CF) was introduced. CF was obtained by dividing the mean value of π_*xy*_(*f, n*) by the mean value of π_*yx*_(*f, n*).

(2) $$\begin{array}{l} \text{CF}=\frac{\frac{1}{n}\sum \pi_{xy}\left(f,n\right)}{\frac{1}{n}\sum \pi_{yx}\left(f,n\right)},\bar{a}=\frac{1}{n}\sum \pi_{xy}\left(f,n\right),\\ \;\bar{b}=\frac{1}{n}\sum \pi_{yx}\left(f,n\right) \end{array}$$

These results were normalized to become a specific set of values leading to the (normalized) factor NF representing the coupling direction. $\max\left(ā,\bar{b}\right)$

(3) $$\begin{array}{l} \text{NF}=\left\{\begin{array}{c} 2,if\;(\max=\bar{a}\wedge \frac{\bar{a}}{\bar{b}}>5)\\ 1,if\;(\max=\bar{a}\wedge 2<\frac{\bar{a}}{\bar{b}}\leq 5)\\ 0,if\;(\max=\bar{a}\wedge 0\leq \frac{\bar{a}}{\bar{b}}\leq 2) \end{array}\right\}\text{and}\\ \text{ NF}=\left\{-\begin{array}{c} -2,if\;(\max=\bar{b}\wedge \frac{\bar{b}}{\bar{a}}>5)\\ 1,if\;(\max=\bar{b}\wedge 2<\frac{\bar{b}}{\bar{a}}\leq 5)\\ 0,if\;(\max=\bar{b}\wedge 0\leq \frac{\bar{b}}{\bar{a}}\leq 2) \end{array}\right\}. \end{array}$$

#### Coupling Direction

The normalization factor NF determines the direction of all causal links between a set of multivariate time series (*x*_mBBI_ and *y*_fBBI_) as a function of frequency *f* . The NF can take the following values: NF = {−2, −1, 0, 1, 2}. Strong unidirectional coupling is indicated if NF is equal to −2 or 2, bidirectional coupling with the determination of the driver-responder relationship exists if NF is equal to −1 or 1, and a similar influence in both directions and no coupling is present if NF is equal to 0. Here, NSTPDC indices were calculated by applying a window (the Hamming window) of lengths *l*, with *l* = 160 samples and a shift of 40 samples (120 samples overlap between each window).

#### Coupling Strength

In addition to NF, the areas (A_fBBI→mBBI, A_mBBI→fBBI, A_fBBI→fBBI, A_mBBI→mBBI, [a.u.]) were determined for identifying the coupling strength using a trapezoidal numerical integration function to approximate the areas generated in space by a coupling factor (CF) values (one CF in each window). These four area indices take values from the range of [0,1]. e.g., A_fBBI→mBBI = 1 indicates that all causal influences originating from fBBI (fetus) are directed toward mBBI (mother), A_fBBI→mBBI = 0 indicates that fBBI does not influence mBBI. Thereby, arrows (→) indicating the causal coupling direction from one time series to the other one, e.g., mBBI←fBBI, indicates the causal link from fBBI to mBBI. Thus, A_fBBI→mBBI represents the causal coupling strength for the causal link from fBBI to mBBI (fetus to mother), A_mBBI→fBBI represents the causal coupling strength for the causal link from mBBI to fBBI (mother to fetus), and A_mBBI→mBBI/ A_fBBI→fBBI represents the causal coupling strength from mBBI to mBBI or mBBI to mBBI [mother to mother/ fetus to fetus (auto-coupling)]. A normalization procedure (zero mean and unit variance) of the time series fBBI and mBBI and scale-invariance were applied (Schulz et al., 2015).

### Surrogate Data

The surrogate data approach (Schreiber and Schmitz, 2000) was applied to test the significance of maternal–fetal HRV and couplings (mBBI-fBBI) between the two groups. Twenty independent surrogate time series were created from the original mBBI and fBBI time series to test for an impaired HRV and coupling in abnormal fetuses. For HRV analyses surrogates were generated by randomly permuting the samples of the original series in temporal order. Different permutations were used for the original series, so that any temporal structure was destroyed in the surrogate time series. For coupling analyses (NSTPDC), additionally, we created 20 uncoupled isospectral isodistribution pairs (iterative amplitude adjusted Fourier Transform (IAAFT) surrogates) from the original mBBI and fBBI time series to preserve linear properties to test linear couplings in the frequency domain (applying NSTPDC). These surrogate data have the same frequency distribution and power spectra as the original pairs of signals but were completely uncoupled (Nollo et al., 2002).

With this statistical definition of a threshold level, finding a coupling in the original time series higher than the threshold leads to reject the null hypothesis, and to detect the presence of a significant coupling. The significance threshold *t*_*su*_ calculated for normal_surrogates_ and abnormal_surroagtes_ was then set as the mean+2^*^SD of the resultant distribution (Stankovski et al., 2017). Thereby, we exclude that the NSTPDC erroneously estimated a certain degree of coupling in the original series, because of estimation bias associated with spectral features were not more present in the surrogate data.

Moreover, the non-parametric Mann-Whitney *U*-Test was applied to determine differences between normal_surrogates_ and abnormal_surroagtes_, considering statistical significant at *p* < 0.01. If coupling values were higher than the corresponding surrogate threshold *t*_*su*_ and no significant differences between normal_surrogates_ and abnormal_surroagtes_ were presented couplings were considered as statistically valid.

### Statistical Analysis

Spearman correlation was applied to test correlation of NF, A_fBBI→mBBI and A_mBBI→fBBI vs. gestational age. Lilliefort test was used to check normality of the features in the two groups. For normally distributed features 1-way ANOVA test was used while Kruskal-Wallis was applied on the features that failed the Lilliesfort test. The significance level was set to *p* < 0.05. All results were presented as mean ± SD. Spearman's correlation coefficients between the coupling features and the HRV features were also estimated.

## Results

**Figure 1:**
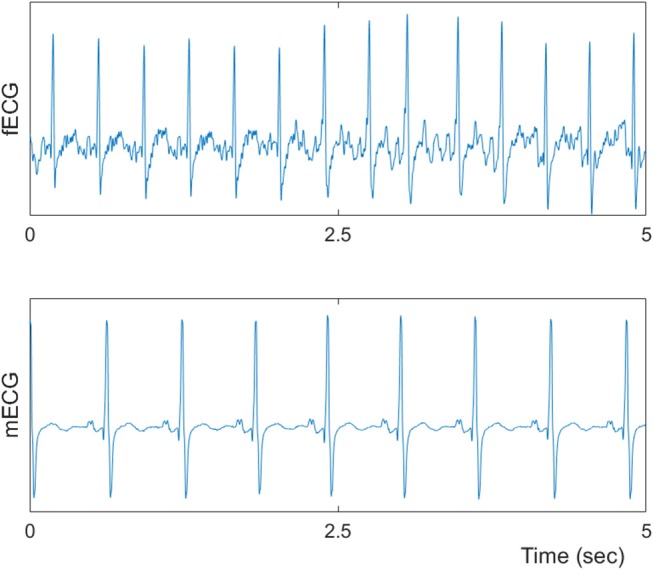
A Sample of fetal and maternal ECGs.

**Figure 2:**
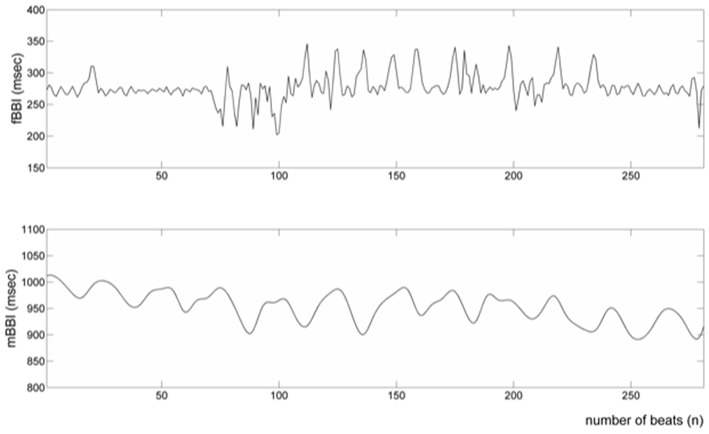
Processed fetal and maternal heart rates (fBBI and mBBI).

Tables 1, 2 summarizes demographics of normal and abnormal fetuses. Ten abnormal cases had some types of CHD (ID: 8, 9, 11, 12, 14–19), two with AV block (ID: 8, 17) and two with Tachycardia (ID: 6, 13). Four cases have abnormalities that were not related to the heart (ID: 2–5). Results of The HRV and NSTPDC analyses are shown in Table 3. Examples of the fetal and maternal ECGs and their corresponding heart rates fBBI and mBBI are shown in Figures 1, 2, respectively.

**Table 3:** HRV and NSTPDC features for the normal and abnormal groups.

	**MeanHr (bpm)**	**sdNN (ms)**	**RMSSD (ms)**	**pNN50 (%)**	**pNNl30 (%)**	**Renyi025**	**Shannon**	**plvar10**	**phvar10**	**LZ77w3b3**	**NF**	**A_fBBI→ mBBI**	**A_mBBI→ fBBI**
**NORMAL**													
mean ± *SD*	145.7 ± 8.9	11.3 ± 6.4	3.1 ± 1.9	3E-4 ± 0.002	1 ± 0.003	2.62 ± 0.65	2.23 ± 0.63	0.92 ± 0.12	2E-4 ± 0.001	0.39 ± 0.06	−0.21 ± 0.85	0.44 ± 0.13	0.46 ± 0.12
Surrogates	145.9 ± 8.9	11.05 ± 6.03	15.63 ± 8.51	0.02 ± 0.06	0.91 ± 0.13	2.61 ± 0.63	2.21 ± 0.62	0.15 ± 0.23	0.03 ± 0.05	0.55 ± 0.09	−1.02 ± 1.06	0.12 ± 0.09	0.31 ± 0. 17
**ABNORMAL FETUSES**													
mean ± *SD*	134.3 ± 38.8	21.5 ± 26.5	13.9 ± 18.5^†^	0.04 ± 0.12^†^	0.92 ± 0.17^†^	3.2 ± 1.2	2.69 ± 1.27	0.65 ± 0.34^†^	0.05 ± 0.13^†^	0.51 ± 0.18^†^	−1.66 ± 0.77^†^	0.08 ± 0.12^†^	0.66 ± 0.24^†^
Surrogates	134.3 ± 38.7	21.55 ± 26.51	30.46 ± 37.5	0.12 ± 0.2	0.79 ± 0.26	3.2 ± 1.2	2.69 ± 1.26	0.19 ± 0.31	0.13 ± 0.19	0.59 ± 0.18	−1.50 ± 0.78	0.08 ± 0.07	0.32 ± 0.12

HR, heart rate in bpm; sdNN, standard deviation of normal fBBI; RMSSD, root mean square of successive differences; pNN50, percentage of normal fBBI interval differences >50 ms; pNNl30, percentage of normal fBBI interval differences < 30 ms; Renyi025, Renyi entropy of the fBBI histogram with α = 0.25; Shannon, Shannon entropy of the fBBI histogram; plvar10, portion of low-variability patterns within the fBBI time series (< 10 ms); phvar10, portion of high-variability patterns within the fBBI time series (>10 ms); LZ77w3b3, Compression entropy with buffer size b = 3 and the window length w = 3; NF, Normalization factor; A_fBBI→ mBBI, coupling strength for the causal link from fBBI (fetus) to mBBI (mother); A_mBBI→ fBBI, coupling strength for the causal link from mBBI (mother) to fBBI (fetus).

† *significantly different from normal group (mean±SD) with p < 0.01. Surrogate analyses did not reveal statistically significant differences*.

### Heart Rate Variability Analysis

The non-linear features were more significant in showing differentiation between the normal and abnormal groups compared to linear features. rmssd and pNN50 were significantly higher for the abnormal group while pNNI30 was significantly lower. The mean and SD (linear features) showed no significant differences. The low variability patterns (quantified by plvar10) were significantly lower for the abnormal group while the high variability patterns (phvar10) were significantly higher. The compression entropy was also significantly higher for the abnormal group.

### Coupling Analyses

**Figure 3:**
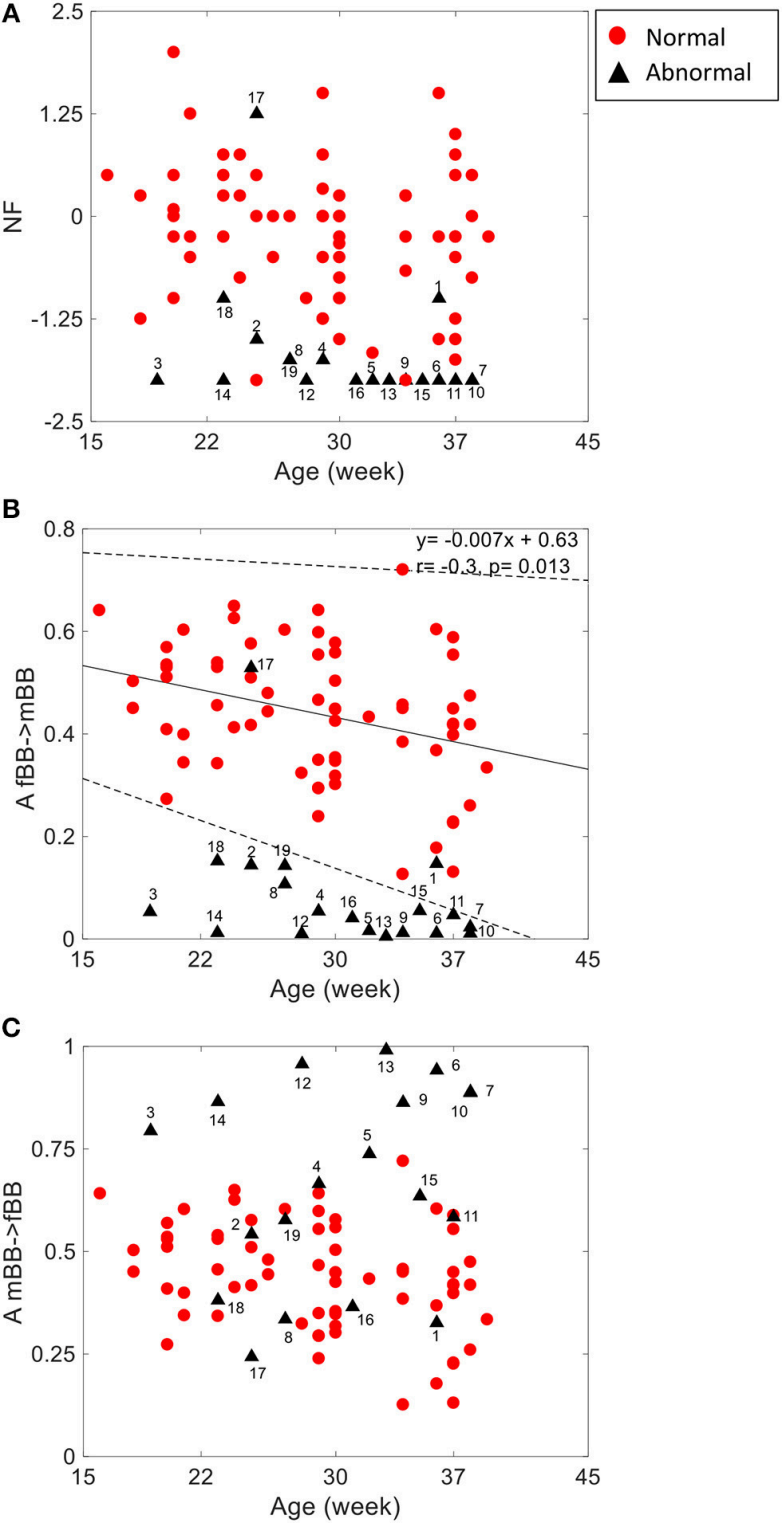
NSTPDC indices (NF: Normalization factor) **(A)**, A_fBBI 1: N indicates the causal coupling strength originating from fBBI (fetus) toward mBBI (mother) **(B)** A_mBBI→fBBI indicates the causal coupling strength originating from mBBI (mother) toward fBBI (fetus) **(C)** vs. gestational age. The numbers indicate the ID of the abnormal cases. The coupling from fetus to mother for the normal group **(B)** showed a significant correlation with age (*r* = −0.3, *p* = 0.013). Solid line represents linear fit for the normal group and dashed lines indicate 95% confidence interval.

For the coupling direction (Table 3), NF was significantly lower for the abnormal. Most of the abnormal cases were having NF≤-1.5 except for three cases (ID: 1, 17, 18, Figure 3A).

For the coupling strength, A_fBBI→mBBI was significantly lower for the abnormal group while A_mBBI→fBBI was significantly higher. Also, all except ID: 17 had A_fBBI→mBBI < 0.2 (Figure 3B) for A_mBBI→fBBI, 10 cases were >0.75 including the cases with Tachycardia (ID: 6, 13) while cases with AV block (ID: 8, 17) were below 0.4 (Figure 3C). A_fBBI→mBBI for the normal group significantly correlates with the gestational age while it was not significant for the abnormal case although it has the tendency to drop with age (Figure 3B).

**Figure 4:**
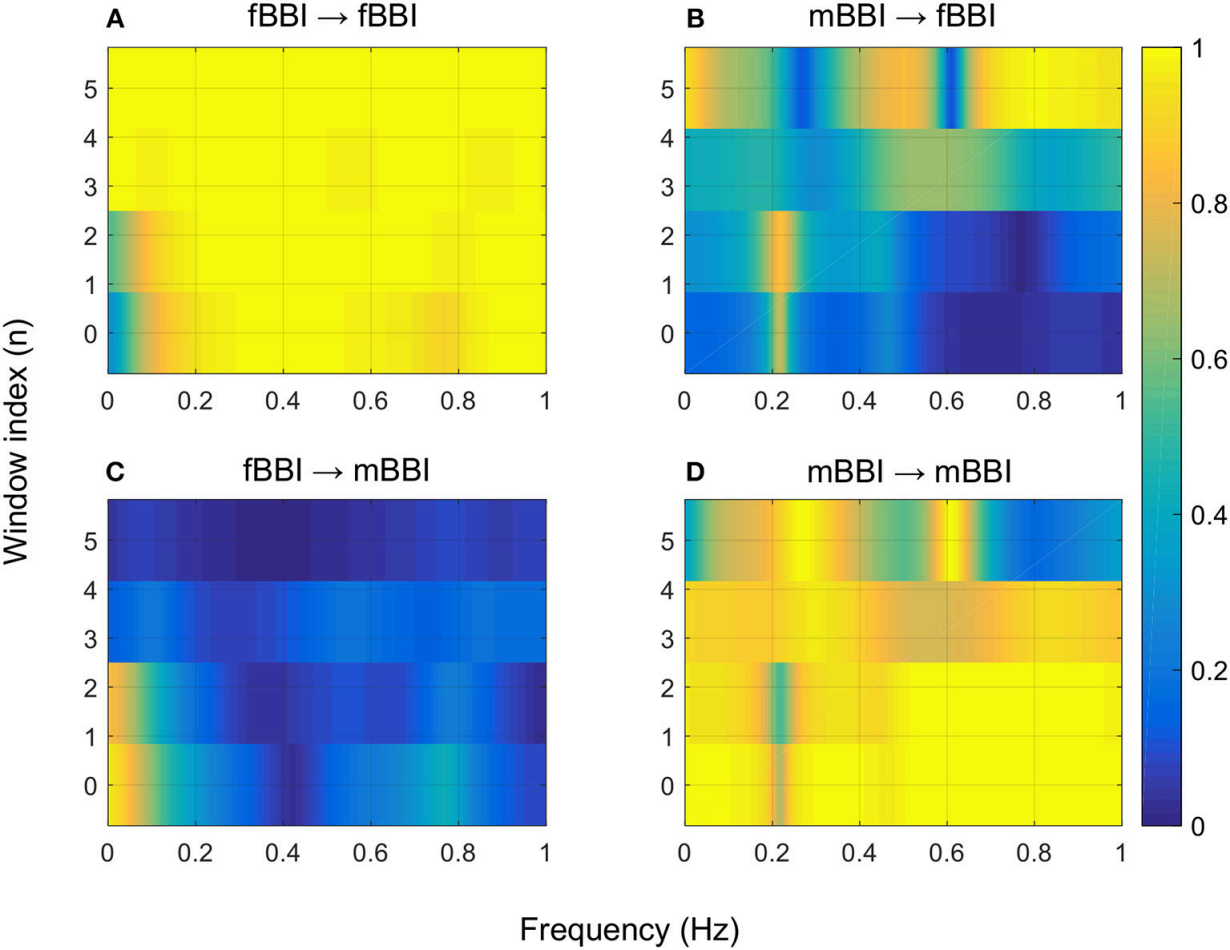
2D NSTPDC plots for: **(A)** fetal auto-coupling (fBBI→fBBI), **(B)** maternal-fetal coupling (mBBI→fBBI), **(C)** fetal-maternal coupling (fBBI→mBBI), and **(D)** maternal auto-coupling (mBBI→mBBI) for a normal fetus with age: 30 weeks. The window length is 160 samples.

**Figure 5:**
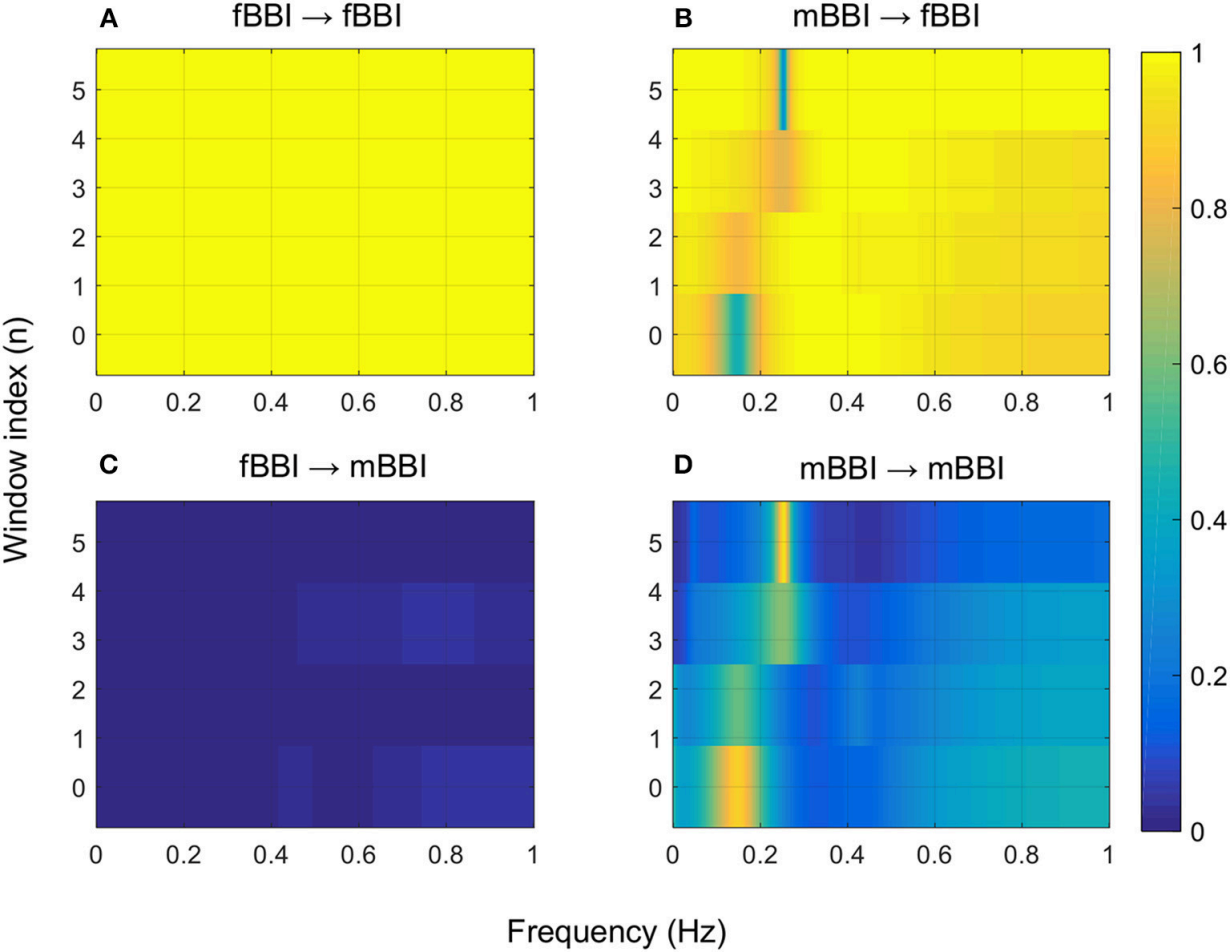
2D NSTPDC plots for: **(A)** fetal auto-coupling (fBBI→fBBI), **(B)** maternal-fetal coupling (mBBI→fBBI), **(C)** fetal-maternal coupling (fBBI→mBBI), and **(D)** maternal auto-coupling (mBBI→mBBI) for a fetus with tachycardia (ID: 13 from Table 2). The window length is 160 samples.

**Figure 6:**
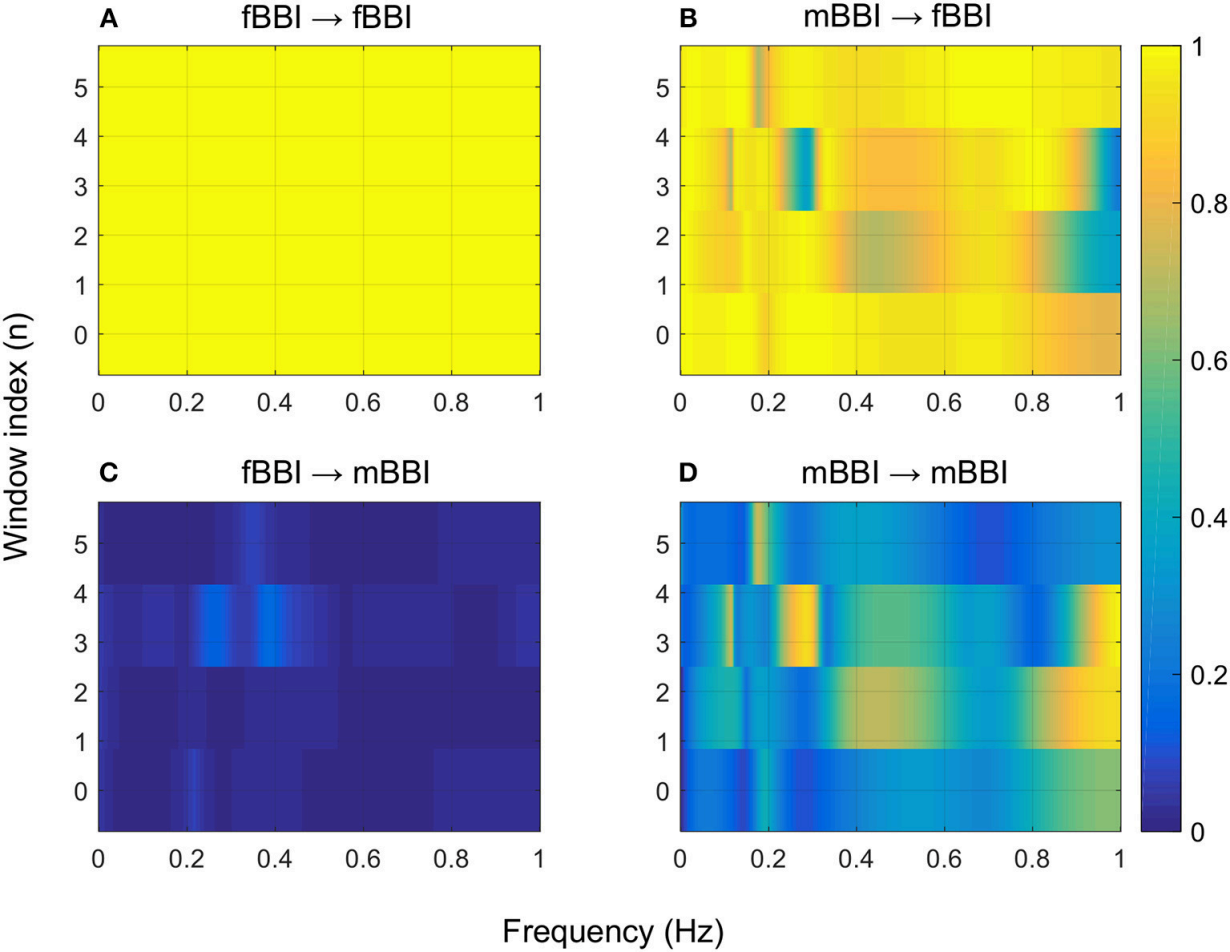
2D NSTPDC plots for: **(A)** fetal auto-coupling (fBBI→fBBI), **(B)** maternal-fetal coupling (mBBI→fBBI), **(C)** fetal-maternal coupling (fBBI→mBBI), and **(D)** maternal auto-coupling (mBBI→mBBI) for a fetus with SSS (ID: 7, Table 2). The window length is 160 samples.

Figures 4–6 show 2D maps of NSTPDC parameters as a function of frequency *f* (160 sample window) for a normal, tachycardia and SSS (Sick Sinus Syndrome) cases. Normal was distinguished from abnormal cases with its high fBBI→mBBI coupling. Although both the tachycardia (ID: 13) and the SSS (ID: 7) had high mBBI→fBBI, the tachycardia showed drop in the mBBI→fBBI coupling around 0.15 Hz and around 0.25 Hz while the SSS case had low mBBI→fBBI in the frequency band 0.8–1 Hz (Figures 5, 6). However, we have to state that couplings higher than half of the mean heart rate (mother) are possibly only caused by “mirrored components” due to cardiac aliasing effects (Milde et al., 2011). This means that a clear physiological interpretation of the results higher than 0.66 Hz is more speculative. A clear relationship of fetal and maternal cardiac coupling in this frequency band is still not yet investigated.

### Surrogate Analyses

Surrogate analyses did not reveal any significant differences between normal fetuses and abnormal fetuses surrogate time series, and thereby, confirming the statistical validity of the found HRV and NSTPDC results (Table 3).

### Correlation Analyses

**Figure 7:**
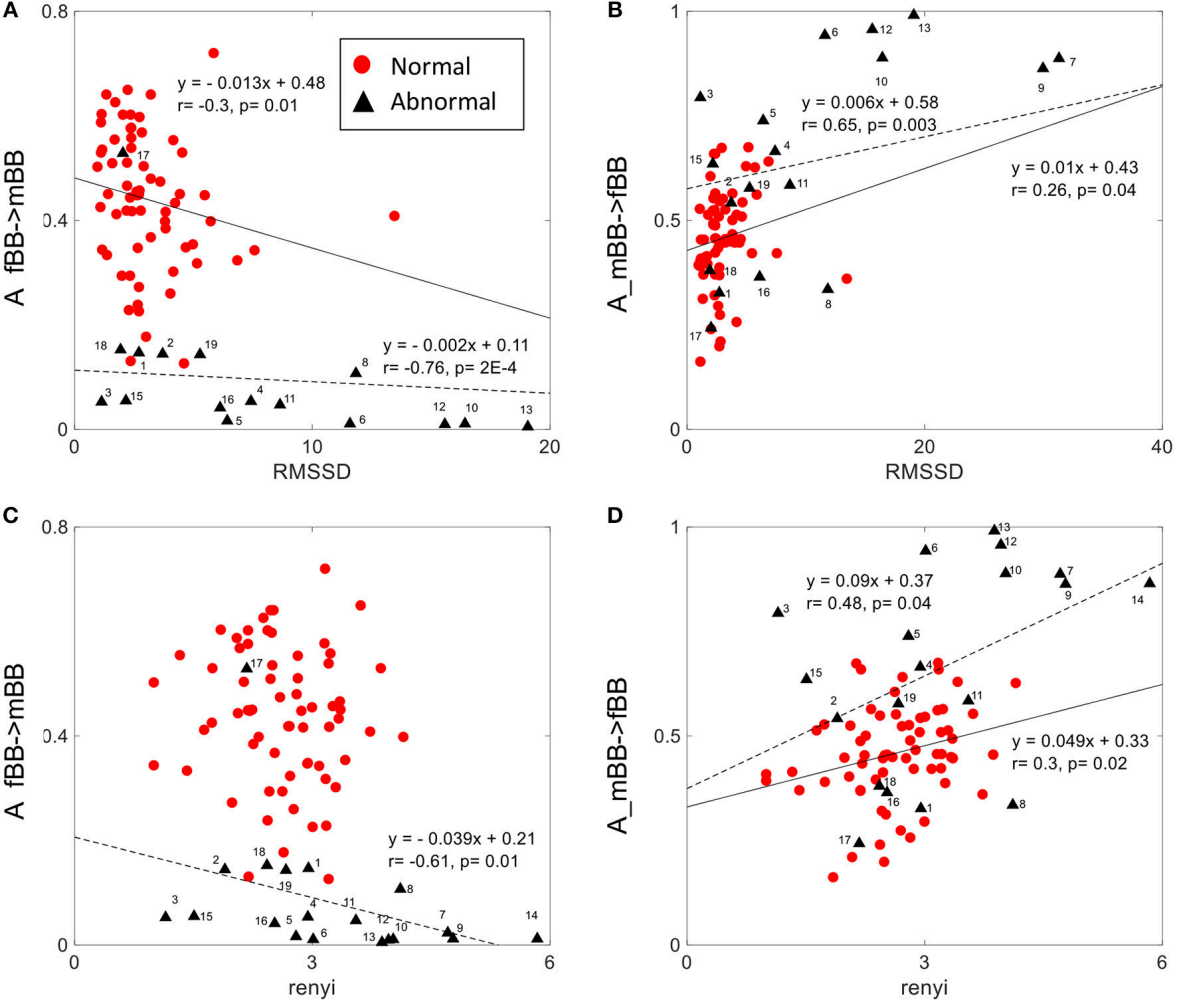
Correlation between coupling parameters (A_fBB →mBB and A_mBB →fBB) and RMSSD **(A,B)** and Renyi entropy **(C,D)** for normal and abnormal groups (solid line represents linear fit for normal, dashed line represents linear fit for abnormal group).

The normalized factor (NF) did not show correlation with any of the other HRV features. A_ fBBI→mBBI and A_mBBI→fBBI had correlation with only the RMSSD and Renyi entropy measures (Figure 7). For normal cases, A_ fBBI→mBBI had weak correlation with RMSSD (*r* = −0.3, *p* = 0.01) while A_mBBI→fBBI had weak correlation with both RMSSD and Renyi entropy (*r* = 0.26, 0.3, and *p* = 0.04, 0.02, respectively). The correlation became reasonably strong in the abnormal cases (Figure 7).

## Discussion

In this study we have evaluated the direction and strength of maternal-fetal heart rate coupling by NSTPDC in normal and abnormal cases as a follow up to our previous study (Khandoker et al., 2016) on normal fetuses. As compared to normal fetuses, abnormal group showed weaker influence of fetal heart rate on maternal heart rate (A_fBBI→mBBI) while the influence of maternal heart rate on fetal heart rate (A_mBBI→fBBI) for the abnormal group was higher.

### Heart Rate Variability Analysis

In abnormal cases, non-linear fetal HRV parameters based on symbolic dynamics were shown to have more significant differences from normal ones. Among symbolic HRV parameters, lower plvar10 [i.e., a decrease in the number of short-term, low-variability episodes (shorter than 10 ms)] and higher phvar10 [i.e., an increase in the number of short-term, high-variability episodes (longer than 10 ms)] in abnormal group might indicate that both branches of autonomic nervous system (sympathetic and parasympathetic) could have been affected. It could also be argued that lower plvar10 and higher phvar10 of beat to beat heart intervals to shift the balance between sympathetic and parasympathetic activity as it was previously reported in adult HRV study (Seifert et al., 2018).

### Fetal-Maternal Coupling Analyses

The group of abnormal cases regardless of gestational age revealed strong unidirectional coupling (NF~-1.7) (Figure 3A) with the maternal heart rate as the driver in comparison to the normal cases (GA2) that showed a similar influence in both coupling directions (NF~-0.2), but with maternal heart rate as driver. In general, the coupling strength for the normal cases from the fetus to the maternal heart rate (A_fBBI→mBBI = 0.44 ± 0.13) was much higher than for the abnormal cases (A_fBBI→mBBI = 0.08 ± 0.12), on the other hand, the coupling strength for the healthy cases from the maternal to the fetus heart rate (A_mBBI→fBBI = 0.46 ± 0.12) was lower than for the abnormal cases (A_mBBI→fBBI = 0.66 ± 0.24) (Figure 3B).

Gradual diminishing causal influence from fetal to maternal heart rate coupling strength over increasing gestational ages (as shown in Figure 3B) was also reported in our previous study (Khandoker et al., 2016). However, weaker A_fBBI→mBBI and stronger _mBBI→fBBI strengths in abnormal cases found in this study could indicate different coupling patterns in placental gas exchange. This was shown by NF that reduces from −1.0 to lower values from normal to abnormal ones indicating that a bi-directional coupling between fetal and maternal heart rate remains to exist while the maternal heart rate acts as the driver (master) in most of the abnormal cases.

For some abnormal cases, the maternal to fetal heart rate influence (A_mBBI→fBBI) was higher than normal while other abnormal cases had similar or lower values than the normal ones. For example, the two cases with cardiac tachycardia were having A_mBBI→fBBI>0.9 while three cases with heart conduction problems (AV block and WPW) were having A_mBBI→fBBI <0.35 (Figure 3C). There is a trend for a drop in fetal to maternal influence and an increase in maternal to fetal influence as the fetus develops over time which is similar to trend found in the normal cases (Figures 1B,C) analyzed in our previous study (Khandoker et al., 2016). However, this needs to be further investigated with a larger sample of abnormal cases.

The 2D color maps of the maternal-fetal coupling were useful visual tools that differentiate various types of abnormalities which had varying coupling strength at different frequency bands (Figures 4–6). For example, the high fetal heart rate (fBBI) in the case of tachycardia might have caused a relatively higher coupling in the high frequency band (Figures 5B,C) while the lower fetal heart rate (fBBI) in the case of SSS, might have caused a shift in the coupling strength to the low frequency band (Figures 6B,C). Although the preliminary findings are interesting, but these observations need to be tested with more cases of similar abnormalities. NF and A_fBBI→mBBI coupling parameters which have shown clear separation of most of the abnormal cases from the normal group (Figures 3A,B) might be useful markers for detecting abnormal fetuses.

The complexity of fetal heart rate was also assessed using different non-linear dynamics features (plvar10, phvar10, LZ77w3b3) which were significant in differentiating between normal and abnormal cases than the traditional linear features (mean, sdNN). The weak or no correlation between the coupling parameter (NF) and linear/non-linear measures of HRV indicates the independency of the proposed coupling strength. However, positive and negative correlations of coupling parameters (A_fBBI→mBBI and A_mBBI→fBBI) and RMSSD and Renyi entropy (Figure 7) could mean that directionality of coupling parameters might be partly associated with fetal parasympathetic (vagal) nervous system. Further studies are required to look at the possible link. The fetal and maternal auto-couplings (fBBI→fBBI and mBBI→mBBI) present the functional states of fetal and maternal autonomic nervous systems, respectively. Fetal and maternal auto-couplings are comparable to autocorrelation functions, looking for repeating coupling structures in one time series. The fetal auto-coupling strength (fBBI→fBBI) is high possibly because the ANS is not completely developed and has low HRV at this age. The maternal auto-coupling strength (mBBI→mBBI) is lower than in fetuses with higher HRV (developing over time) possibly because the most subsystems of ANS are working in a stable manner in this age period.

The actual relationship between interrupted fetal oxygenation through placenta and fetal abnormalities is very complex and incompletely understood. In recent years, it has become apparent that most cases of fetal anomalies are unrelated to intrapartum events and therefore cannot be prevented by intrapartum fetal heart rate (FHR) monitoring. We believe that NSTPDC monitoring of both fetal and maternal heart rates introduced in this study with the expectation that it would significantly reduce the incidence of fetal compromise caused by intrapartum interruption of fetal oxygenation. According to placental physiology, oxygen is transferred from the placenta to the fetus by maternal and fetal blood along a pathway that includes the maternal lungs, heart, vasculature, uterus, placenta and umbilical cord (Simpson and MacDonald, 1981). The most understood mechanism of fetal response to interrupted oxygen transfer (if caused by abnormal rhythms of fetal and maternal heart rates) involves a sequential physiologic progression namely from hypoxemia to hypoxia leading to metabolic acidosis and then consequently metabolic acidemia (Simpson and MacDonald, 1981). We speculate that inability of abnormal fetal heart to adapt the adequate oxygen transfer through placenta could have been reflected on altered A_fBBI-mBBI and A_mBBI-fBBI coupling parameters.

A limitation of the NSTPDC approach in the case of fetal-maternal cardiac coupling is that it is only able to detect linear couplings in the frequency domain. Thus, couplings higher than half of the mean heart rate (mother) are probably “mirrored components” due to cardiac aliasing effects (Milde et al., 2011), and therefore, not of physiological relevance. That means there is a lack of physiological components at frequencies higher than half of the mean hear rate of the mother (mBBI). For instance, mean maternal heart rate is 80 bpm (1.33 Hz, which corresponds to a Nyquist frequency of 0.66 Hz) the physiological meanings of the auto-couplings (mBBI→fBBI, mBBI→mBBI) within the band of 0.66–1.0 Hz are unclear.

In conclusion, the results of NSTPDC analysis in this study provided more details about the direction and strength of the causal link between maternal and fetal heart rates and their changes with the types of fetal abnormalities as compared to normal fetuses. These non-linear and coupling methods between fetal and maternal heart rate could help monitor the development of normal fetal cardiac health and identify pathologies throughout the pregnancy period before delivery and thus could initiate preemptive actions to save tiny lives *in utero*. However, fetal endocrinal and biophysical research are required to verify our understanding of the physiological mechanisms of transplacental gas transfer, and the ways in which mathematical modeling emulates those processes.

### Study Limitations

Duration of the collected ECGs was 1–2 min which is typical in routine visits during pregnancy. However, longer duration of 5–10 min will be needed for better evaluation of coupling and HRV measures on longer term. Also, more cases of similar types of abnormality are required to do a quantitative analysis between the types of abnormality and the values of these features. A limitation of the NSTPDC approach is that the physiological meanings of the auto-couplings (mBBI→fBBI, mBBI→mBBI) within the band of 0.66–1.0 Hz are unclear.

## Approval, Accordance, and Informed Consent

The study protocol was approved by Tohoku University Institutional Review Board (IRB: 2015-2-80-1) and written informed consent was obtained from all subjects. All experiments were performed in accordance with relevant guidelines and regulations.

## Author Contributions

AK, SS, and AV designed the study. YK collected the maternal and fetal ECG data. SS and AV applied signal processing methods and extracted coupling features. SS and HA-A ran statistical analysis on results. AK, SS, HA-A, and AV evaluated results of the statistical analysis. HA-A and AK wrote the main manuscript text and prepared the tables and figures and SS revised them. All authors reviewed the manuscript.

### Conflict of Interest Statement

The authors declare that the research was conducted in the absence of any commercial or financial relationships that could be construed as a potential conflict of interest.
